# An accelerated human in-vitro aging model mimicsin-vivo aging and facilitates dynamic testing of anti-aging compounds

**DOI:** 10.21203/rs.3.rs-6173768/v1

**Published:** 2025-03-28

**Authors:** Jerome Mertens, Larissa Traxler, Lukas Karbacher, Oliver Borgogno, Taylor Ozbun, Kylie Champion, Anna Smaling, Barbara Boeckle, Hildegard Mack, Michaela Defrancesco

**Affiliations:** UCSD; University of California San Diego; University of California San Diego; University of California San Diego; University of California San Diego; University of California San Diego; University of Innsbruck; Medical University of Innsbruck; University of Innsbruck; Medical University of Innsbruck

**Keywords:** Aging, Longitudinal model, disease model, anti-aging treatments, heterogeneity, biological age, epigenetic clocks, accelerated aging, direct conversion, induced neurons

## Abstract

Biological aging drives cellular dysfunction and human disease, yet studying human-specific aging dynamics remains challenging due to limited experimental platforms. Here we show that long-term post-mitotic culture of human fibroblasts authentically recapitulates and accelerates in-vivo aging signatures. Longitudinal paired transcriptomic-epigenetic analyses revealed that in-vitro aging mirrors in-vivo primary fibroblasts aging, with concordant transcriptional aging pathways and accelerated epigenetic clock aging patterns. Direct neuronal conversion of pre-aged fibroblasts preserved biological age, enabling pseudo-longitudinal modeling of neuronal aging. Single-cell transcriptomics revealed a time-dependent increase in age-heterogeneity, reflecting in-vivo observations and revealing heterogeneity driven by the variable loss of transcriptional programs. Using this accelerated aging platform, we evaluated anti-aging compounds: Metformin broadly halted transcriptomic and epigenetic aging, while Rapamycin showed limited efficacy. These findings align with clinical evidence, demonstrating our platform’s capacity to predict therapeutic anti-aging efficacy with molecular resolution. This system advances our understanding of aging mechanisms and facilitates the development of interventions against age-related diseases.

## INTRODUCTION

Biological aging leads to decreased cellular functionality, and an increase in organismal dysfunctions, illnesses, and mortality. Due to unique human genetics, physiology, and lifespan, and the human specificity of many aging-related disorders, the predictive value of animal models is limited, but models to authentically model longitudinal human cellular aging are lacking. Human primary cell cultures and direct reprogramming technologies, such as induced neurons, have enabled the generation of donor- and patient-specific cell models that closely resemble somatic cells and tissues^1–5^. However, while age-equivalent models capture authentic human aging signatures of a donor at a given age, this cross-sectional approach does not provide insights to the dynamic aging process or offer means to test intervention strategies against it. To trigger specific aging pathways in cell models, stressors such as chemical treatments including oxidative agents^6^, proteosome inhibition or senescence-induction^7,8^, or overexpression of aging-related factors such as progerin^9,10^ have been applied, but they are limited to the extent they can authentically recapitulate the width and complexity of cellular aging. Meanwhile, the overall rate of aging appears to be strictly linked to the progression of time – typically the chronological age of an organism – and models that recapitulate many aspects of the time-dependent human aging process would be very valuable. Currently however, it remains unknown how biological aging is modulated during long-term human cell culture, and in particular in non-replicative culture settings without clonal selection or replicative exhaustion/senescence, which would be more similar to the in-vivo tissue situation.

In this study, we present a model system to induce aging in human primary fibroblasts in-vitro, authentically mimicking in-vivo aging without triggering replicative senescence. By culturing fibroblasts at confluency for six months, we achieved significant molecular aging, including an 8-year gain in DNA methylation age. Single-cell transcriptomic analyses revealed an increasing heterogeneity in cellular aging over time, driven by the variable downregulation of specific transcriptional programs. This heterogeneity reflects in-vivo aging dynamics and highlights the asynchronous nature of cellular aging within populations. This approach offers a valuable tool for investigating the critical time windows of age-related disease onset. Importantly, as fibroblasts serve as the starting material for direct conversion protocols, which retain age-related signatures of the original cells, this system facilitates pseudo-longitudinal studies of age-related diseases across various tissues. We here demonstrate that pre-aged fibroblasts can generate induced neurons that preserve their biological age, enabling modeling of neuronal aging. Using this platform, we assessed the effects of two candidate anti-aging compounds. Metformin broadly arrested transcriptomic and epigenetic aging, while Rapamycin showed limited efficacy. These results align with existing clinical findings, underscoring the utility of this platform for predicting therapeutic outcomes. Our work establishes a robust framework for studying human aging mechanisms and developing interventions for age-related diseases.

## RESULTS

### Long-term non-dividing in-vitro culture recapitulates and accelerates human fibroblast aging

**Figure 1:**
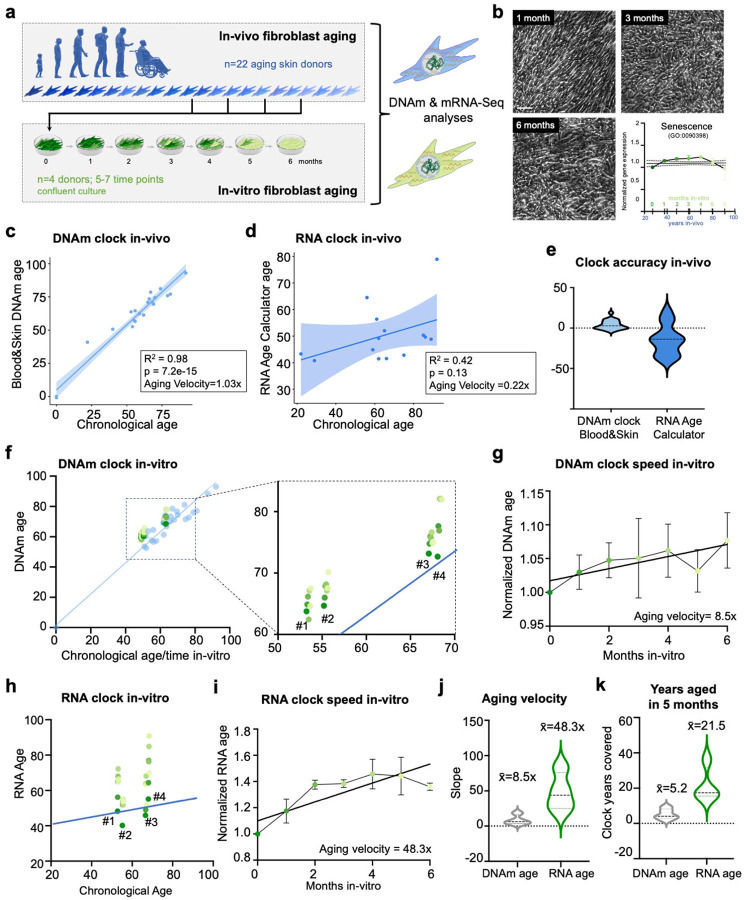
Epigenetic and transcriptomic clock comparing in-vivo and in-vitro aging. (a) Human fibroblasts of donors aged 0–92 years were harvested once reaching confluency (in-vivo aging). Donors aged 53, 55, 67, and 68 were grown confluent (time-point 0) and kept in culture without splitting for six months. RNA-Seq and DNAm EPIC Array was performed at 5–7 time-points each 30 days. (b) Phase contrast images taken at time of harvest each month. Gene expression related to Senescence GO Term. (c) Epigenetic Blood&Skin DNAm Age was calculated using the Online New Methylation Age Calculator for the in-vivo of donors between 0 and 92 years of age (n=22). (d) RNAAgeCalc (RNA clock) without tissue specialization was applied to fpkm values of the in-vivo aging transcriptomic dataset (n=14). (e) The difference between estimated age and chronological age for Blood&Skin DNAm Age and RNA Age. (f) Blood&Skin DNAm Age was applied to methylation data of in-vitro aging. N=4 independent samples. (g) RNA Clock for estimation of transcriptomic age of in-vitro aging dataset. N=4 independent samples. (h) Aging velocity, defined as the slope of the linear regression, was calculated for both DNAm and RNA Age for in-vitro and in-vivo aging. (i) Difference in DNAm and RNA Age of month 5 compared to starting point (month 0).

Here, we set out to explore a long-term non-dividing in-vitro culture as a model for cellular aging that allows for a dynamic investigation of the aging process by mapping and directly comparing in-vivo aging with in-vitro aging signatures within the same cell type using genome-wide DNAm and mRNA-Seq analyses (Fig. 1a). We first obtained primary human fibroblasts from skin punch biopsies from 22 individuals that have lived and aged 0 to 92 years in-vivo. To control for changes that are due to the in-vitro culture environment, the 22 in-vivo-aged cell lines were harvested before reaching passage 10, and assessed at full confluency. From four of the donors, aged 53, 55, 67, and 68 years (**Fig.S1a**), we continued culture at confluency without passaging for six months. Long-term cultures of confluent non-dividing fibroblasts remained stable without apparent morphological changes, areas of overgrowth, or cell death, and in contrast to sequential passaging experiments^4^, they do not enter a senescence-associated state (Fig. 1b). To quantify biological aging signatures, we first employed epigenetic and transcriptomic clock analyses of our in-vivo- and in-vitro-aged samples. Epigenetic clocks are age estimators based on DNA methylation (DNAm) values at combinations of genomic loci, and they have proved very useful to measure and quantify human biological age^11–13^. The DNAm Blood&Skin clock^14^ outperformed the Hannum clock^12^, and the PhenoAge clock^13^ in cultured human fibroblasts, showing a near perfect correlation of DNAm age to the chronological age of the 22 in-vivo-aged donor samples (R^2^ = 0.98, p = 7.2^− 15^; Fig. 1c). The Blood&Skin clock only minimally overestimated chronological age with a velocity of 1.03x, while the other tested clocks underestimate chronological age, and showed weaker correlation to chronological age (Hannum: 0.40x, PhenoAge: 0.48x; **Fig.S1b-c**). We thus continued to use the Blood&Skin DNAm clock to assess DNAm ages in this study. However, while stunningly accurate, epigenetic clocks utilize DNAm sites in the genome that are rarely associated with genes, rendering their biological interpretation difficult^15^. By contrast, transcriptome signatures are more directly related to cellular functions, and meta-analysis of transcriptional aging data revealed that chronological age can also be predicted based on mRNA-Seq data^16^. RNA clocks utilize genes that participate in hallmark processes of aging, such as proteasome and ribosomes, mitochondrial and metabolic pathways, inflammation, and DNA replication and repair^16,17^. We thus probed for transcriptomic age using the RNA Age Calculator, of which one version is trained on an extensive dataset of different tissues (RNA Age Calculator - no tissue) and one trained on fibroblast data (RNA Age Calculator - Fibroblast)^16^. Applied to our in-vivo-aged cohort, both RNA clock versions ticked slower than chronological time, with an aging velocity of 0.22x (no tissue) and 0.13x (fibroblasts). Surprisingly, the RNA Age Calculator - no tissue (R^2^ = 0.42, p = 0.13) was more accurate than the fibroblast-specific version (R^2^ = 0.23, p = 0.44) (Fig. 1d; **Fig.S1d**). Overall, DNAm clock analysis proved to be more accurate than RNA clock analysis, and it only slightly overestimated chronological age by 3.9 years on average (+/− 5.6 years), while the RNA Age Calculator (no tissue) underestimated chronological age by 14.6 years (+/− 18.81 years; Fig. 1e; **Fig.S1e**). We continued with using the RNA Age Calculator (no tissue) for our study.

Next, we followed our in-vitro-aging cohort with monthly DNA/RNA extractions, and applied the clock analyses (n = 4 donors; 4–6 time points per donor). We found that the DNAm clock continued to tick, but at an accelerated pace in-vitro for all donors (Fig. 1f; **Fig.S1f**), with an average velocity of 8.5x (Fig. 1g). Interestingly, also the RNA clock continued to tick in-vitro (Fig. 1h; **Fig.S1g**), but its velocity uncoupled from epigenetic aging and accelerated to an average RNA aging velocity of 48.3x with substantially higher variability across donors (Fig. 1i–j). The average extent of biological age covered within five months in-vitro thus was much higher on the transcriptome than on the epigenetic level, resulting in a net-gain of 5.2 epigenetic years and 21.5 transcriptomic years (Fig. 1k). These data demonstrate that epigenetic and transcriptomic features of in-vivo aging are recapitulated and accelerated in-vitro, and that global transcriptomic aging accelerates to a higher velocity than the epigenetic aging process.

### In-vitro aging authentically recapitulates and accelerates in-vivo transcriptomic signatures

**Figure 2:**
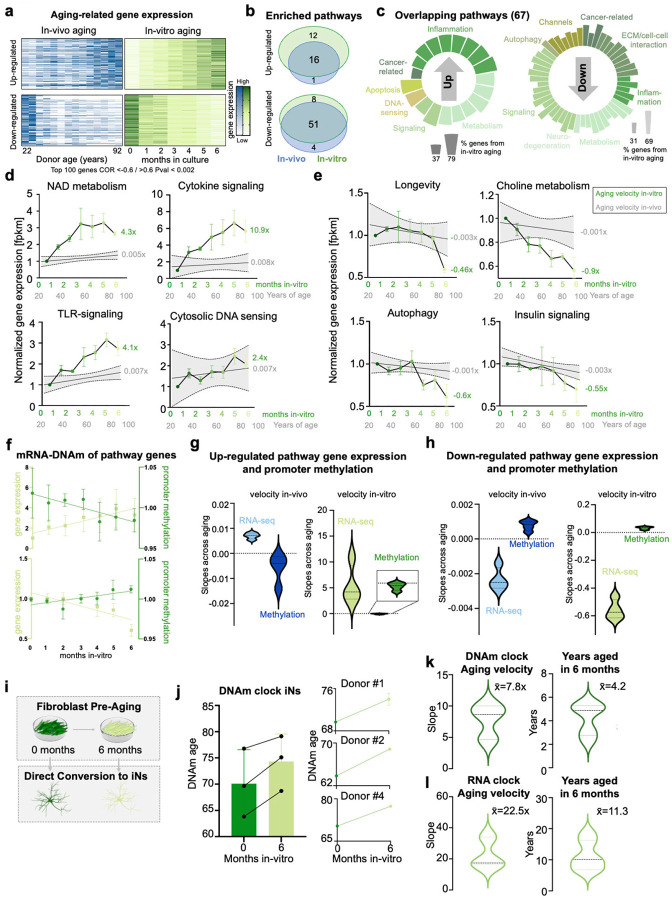
In-vitro aging transcriptome changes are concordant with human fibroblast aging in-vivo. (a) Top100 up- and downregulated genes with significant regression along in-vivo aging (blue) and in-vitro aging (green). (b,c) CLUEGO combined analysis of significant genes involved in in-vivo and in-vitro aging was performed and corrected for size of input list. (in-vitro aging, p<0.05: n=4791 downregulated, 290 upregulated genes; in-vivo aging, p<0.1: n=794 genes, n=347 upregulated genes). Pathways were considered overlapping when showing a cluster gene contribution of 30–70 %. (d,e) Selected KEGG pathways overlapping gene expression along time comparing in-vivo aging (grey) and in-vitro aging (green). All values were normalized z-score and average per donor is plotted. Aging velocity (slope of Linear Regression) is shown as number next to each pathway. (f-h) Up- and down-regulated genes of selected pathways and their promoter methylation defined as methylation at TSS200, TSS1500, 5’UTR, and 1^st^ exon over time and their slopes in-vivo (blue) and in-vitro (green). (i-k) Fibroblasts were pre-aged with the established protocol and converted to induced neurons at baseline and after 6 months of in-vitro aging. Blood&Skin DNAm clock was calculated (n=3 donors, 2 replicates for 2 donors at month 6). Linear Regression was performed to assess aging velocity. Years aged was calculated by subtracting estimated age at baseline from month 6. (l) Aging velocity and years aged in 6 months based on transcriptomic age of RNA Age Calculator (no tissue).

We asked to what extent in-vitro aging recapitulates authentic in-vivo signatures of cellular aging. Because the RNA clock utilizes expression values of genes across multiple biological functions, we next sought to assess the transcriptional overlap of individual biological processes that age both in-vivo and in-vitro. Pearson correlation on gene expression changes with years of age (in-vivo-aged samples) and time in culture (in-vitro-aged samples) identified 137 upregulated and 289 downregulated genes significantly correlating to in-vivo aging, and 4791 downregulated genes and 242 upregulated genes significantly correlating to in-vitro aging (p < 0.05, Fig. 2a). The lower numbers of significantly correlated genes in-vivo may be likely in part due to varying genetic backgrounds. Significant and commonly down-regulated aging genes include known and previously validated age-associated genes such as the metabolic regulators SIRT1 and NQO1, the transcription factor CREB3L2 involved in unfolded protein response, and the nuclear pore protein RanBP17, and genes involved in inflammation and skin aging such as JUN, IRF7 and CD40 were significantly and commonly up-regulated^2,18–22^ (**Fig.S2a,b**). Importantly, we found that 93% of all significantly enriched in-vivo aging pathways were authentically recapitulated in-vitro. Specifically, 16 of the 17 up-regulated, and 51 of the 55 down-regulated KEGG pathways (KEGG) that were significantly enriched within in-vivo aging genes were also significantly enriched in aging genes in-vitro changing into the same direction (Fig. 2b). The overlap of individual genes between in-vitro and in-vivo was smaller than the overlap on the pathway level (**Fig.S2c**). Further, only 5 of the 87 significant in-vitro aging pathways were not detected in-vivo, indicating that, surprisingly, culture artifact-related ‘false-positive’ changes remain relatively small. Both common up- and down-regulated signatures included metabolic, signaling, inflammation, and cancer-related pathways, while up-regulated genes were additionally enriched for DNA-sensing, apoptosis pathways, neurodegeneration, channel- and ECM/cell-cell interaction pathways (Fig. 2c). The relative contribution of in-vitro and in-vivo aging genes ranged between 37% and 79% per pathway. Interestingly, and in consistence with the observed RNA clock acceleration, several up-regulated pathways with similar contribution of in-vitro and in-vivo aging, were accelerated several-fold in-vitro, such as NAD metabolism, cytokine signaling, and TLR-signaling (Fig. 2d). Other up-regulated pathways such as cytosolic DNA sensing were more moderately accelerated compared to the in-vivo situation. The down-regulated pathways such as longevity, choline metabolism, autophagy, and insulin signaling showed a stark decline in gene expression with both in-vivo and in-vitro aging (Fig. 2d–e). To assess how promoter methylation (DNAm) of aging genes correlates with mRNA expression, we first selected all genes associated with the overlapping pathways of in-vitro and in-vivo aging and measured their promoter methylation^23^. As expected, and reassuringly, promoter DNAm and gene expression were inversely correlated in-vitro and in-vivo (Fig. 2f). We found that both epigenetic and transcriptomic aging velocities were substantially slower in-vivo (slope transcriptome: 0.007/−0.002, slope DNAm: −0.006/0.001) compared to in-vitro aging (slope transcriptome: 5.4/−0.56, slope DNAm: −0.06/0.03), and within both groups, transcriptome changes were much more pronounced than DNAm changes (Fig. 2g–h). However, aging pathways were similarly affected on transcriptome and DNAm level, suggesting a strong relationship of epigenetic regulation of aging gene expression also during in-vitro aging (**Fig.S2d**). These data show that in-vitro aging recapitulates and accelerates authentic in-vivo transcriptome signatures, and that transcriptomic aging velocities substantially vary across different pathways. The data further emphasize the notion that while in-vitro transcriptomic aging velocities seem to uncouple from DNAm in terms of velocity, they remain functionally coupled.

### In-vitro pre-aging of fibroblasts generates pseudo-longitudinal induced neurons (iNs) with enhanced aging signatures

Direct conversion of fibroblasts from aging human donors into age-equivalent iNs has been instrumental in studying age-related neuronal phenotypes^24–28^. However, obtaining true longitudinal samples from the same donors over extended periods remains challenging. To test whether in-vitro aged fibroblasts could serve as a starting source for iNs with increased aging signatures, we cultured fibroblasts from three donors for 6 months before converting them to iNs using our established protocol (Fig. 2i). To quantify biological aging signatures, we employed epigenetic and transcriptomic clock analyses at baseline and after 6 months of in-vitro aging. Our results demonstrated that iNs derived from 6-month aged fibroblasts exhibited an average increase of 4.1 ± 1.4 years in DNAm age (Fig. 2j–k), and increase of 11.3 ± 4.8 years in RNA age (Fig. 2l) compared to iNs from non-aged fibroblasts. These data demonstrate that aging signatures acquired by fibroblasts during extended in-vitro culture are preserved during the iN conversion process. This approach provides a viable strategy for creating pseudo-longitudinal iN samples to model progressive neuronal aging in vitro.

### Single-cell RNA-seq reveals increasing cellular heterogeneity and non-uniform aging trajectories during in-vitro fibroblast aging

**Figure 3:**
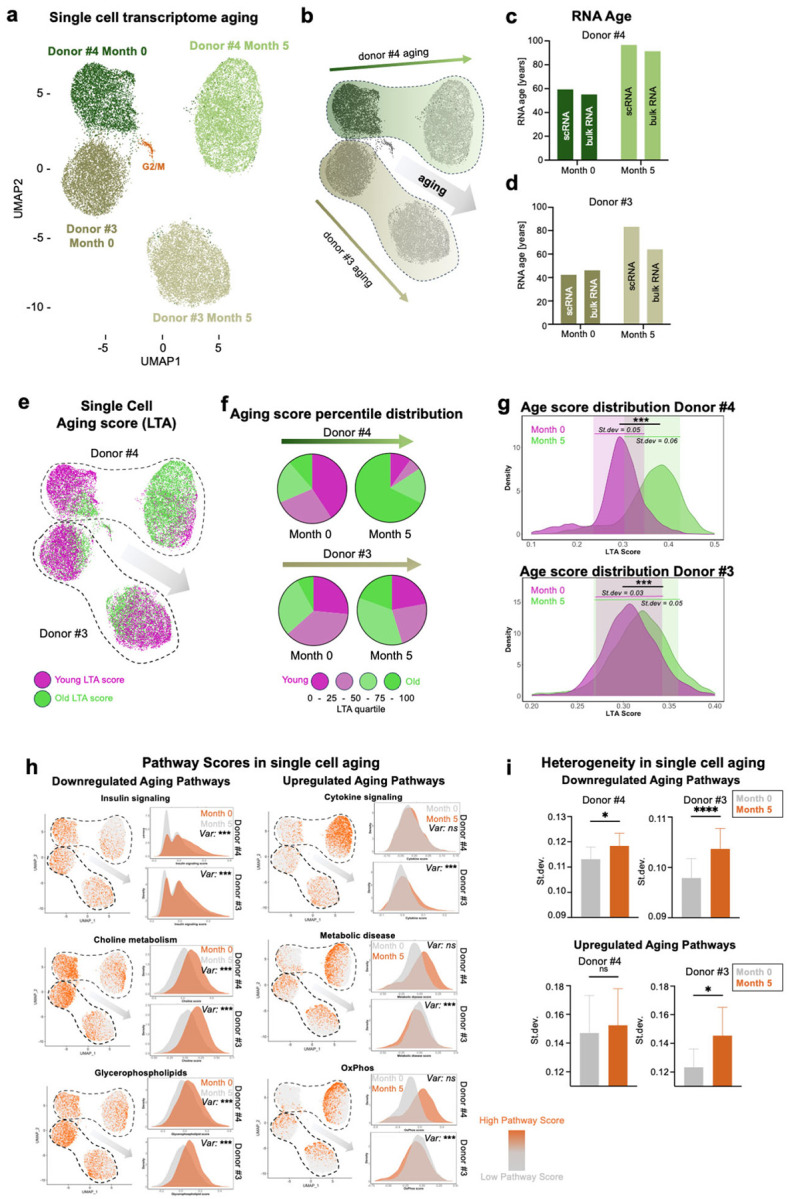
Single cell RNA sequencing shows heterogenous aging in-vitro. (a,b) UMAP of scRNA-seq of Donors #3 and #4 at Month 0 and Month 5. (c,d) RNA Age of the donors at Month 0 and Month 5 of bulk RNA sequencing and pseudobulk of single cell RNA sequencing. (e) UMAP of LTA score based on overlapping aging genes in-vitro and in-vivo, dividing cells into young cells (lower 50 % LTA score, purple) and old cells (upper 50 % LTA score, green). (f) Contribution of cells of each donor and time-point to each quartile. (g) Distribution of single cell LTA score per condition, highlighting Standard deviation for each sample. Levene’s Test for Homogeneity of Variance (#4 Df=1, F value=87; #3 Df=1, F value 114.39, p-value <−2.2e-16) (h) UMAPs of highlighted up- and donw-regualted pathways including density plots showing pathway score distrivution. Var. = Levene’s test for homogeneity of variance. (i) Standard deviation of each up- and down-regualted pathway for Donor #3 and #4. Paired t-test.

To further investigate how these aging signatures manifest across individual cells, we next explored the heterogeneity of the aging process using single-cell RNA sequencing (scRNAseq). The biological aging process of higher organisms is a heterogenous process, but the heterogeneity of cellular age within a population, and how it changes during in-vitro aging, remains unknown^29,30^. We performed scRNAseq of fibroblasts from two donors, aged 67 years (donor #3) and 68 years (donor #4), at month 0 and following five months of in-vitro aging. The samples contained 5,419–8,145 cells per sample that consistently were covered with 23,800 – 32,5000 reads per cell (**Fig.S3a-b**). The virtual absence of cells in G2/M phase further confirmed the non-proliferative state of fibroblasts during long-term confluent culture in all four samples (Fig. 3c). The two donors and time points clearly clustered separately in the UMAP space (Fig. 3a–b). We first assessed the level of consistency between RNA ages (RNA Age Calculator - no tissue) calculated from scRNAseq pseudobulk expression data of the four clusters and the bulk mRNA-Seq results from the same donors and time points. For both donors, we found very similar predicted RNA ages for both data sets, demonstrating both robustness and reproducibility of the approach (Fig. 3c–d). We next sought to assess heterogeneity of in-vitro aging within the cultures. Because RNA Age Calculator cannot be applied to individual cells due to the low coverage obtained from single cell experiments, we developed a longitudinal aging score (LTA score) comprising of all genes contributing to the overlapping in-vitro and in-vivo aging pathways (Fig. 2). Interestingly, already in primary cultures at month 0, we detected a substantial level of heterogeneity of RNA ages across single cells in each culture, while donor #3 was characterized by a higher ratio of young cells and lower ratio of old cells compared to donor #4, suggesting that the primary fibroblast cultures maintain the age-related heterogeneity of donors (Fig. 3e–f). Specifically, a majority of cells at month 0 from both donors contributed to the youngest quartile of the aging score (40%/26%), and 11%/8% of cells contributed to the upper quartile of old cells (Fig. 3f). Following five months of in-vitro aging, the ratios shifted towards smaller proportions of younger cells, and higher proportions of older cells, resulting in 68% (donor #4) and 20% (donor #3) of cells contributing to the oldest quartile (Fig. 3f). We further separately calculated single cell aging scores based on the genes contributing to the RNA Age Calculator, which resulted in similar effects as seen^30–33^ for the LTA score (**Fig.S3d-h**). Both single cell aging scores similarly revealed a significant increase in age-related cell-to-cell heterogeneity, indicated by increase in Standard deviation of scores within one cell population following five months of in-vitro aging (Fig. 3g, **Fig.S3h,i**). Increasing transcriptional and epigenetic noise and cell-to-cell variations is a key feature of biological aging and has been highlighted as a hallmark feature of aging^30–33^. Heterogeneity increased most notably within the downregulated aging pathways, with cell-to-cell variability significantly rising after five months of in-vitro aging (Fig. 3h,i). In contrast, upregulated pathways exhibited a more modest increase in cell-to-cell heterogeneity over time. These findings suggest that age-dependent heterogeneity emerges predominantly through the variable loss of cellular programs rather than through the activation of aging-responsive pathways, and indicate that these downregulated aging pathways may play a primary role in initiating the aging process, while activation of pathways (e.g. inflammatory) in response may often be secondary.

### Metformin, but not Rapamycin, significantly alters aging trajectories in long-term fibroblast cultures

**Figure 4:**
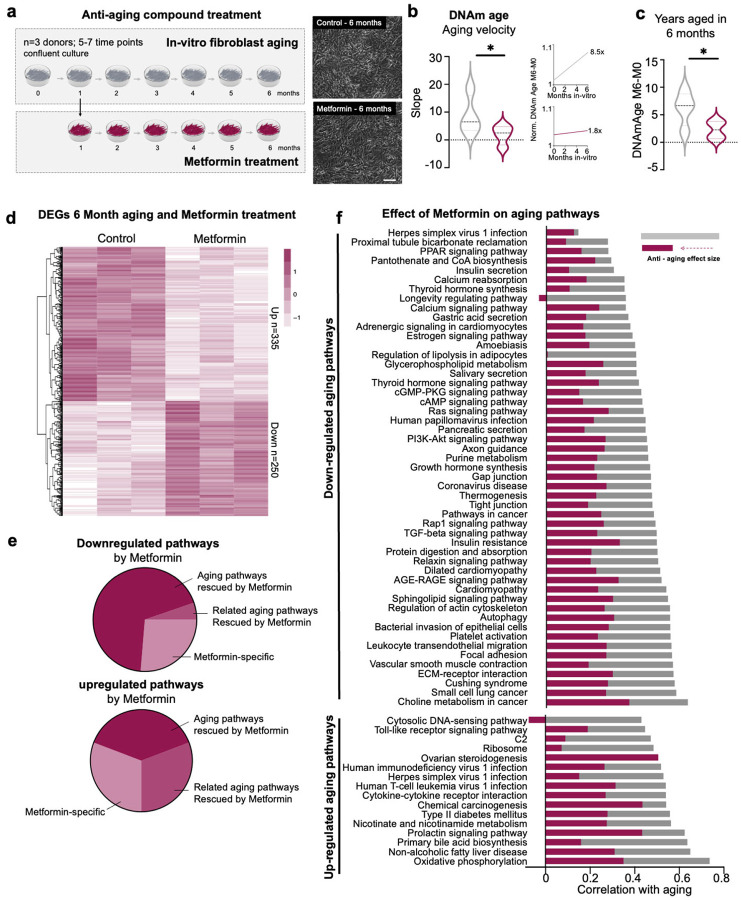
Anti-aging drug Metformin to slow aging in-vitro. (a) Fibroblasts at confluency were treated with Metformin (1mM) for six months (n=3 independent donors). No morphological differences were observed. Cells were harvested each month (30 days) for RNA and DNA extraction to assess epigenetic age (DNAm Blood&Skin clock) and transcriptomic age (RNA Age Calculator - no tissue). (b) DNAm Blood&Skin clock in Control (grey) and Metformin-treated (red) cultures. Paired t-test. Simple linear regression was performed and slopes plotted. (c) Years aged in-vitro was calculated by subtracting the age of Month 0 from Month 6 in Control (grey) and Metformin-treated cultures (red). (d) Differential expression analysis comparing Metformin and untreated control cultures at Month 6. P-adjusted < 0.05. (e) KEGG pathway analysis of DEGs comparing Metformin and untreated cultures, overlapping with aging pathways from (Fig. 2). Dark-red = exact overlaps, medium-red = related pathways, no exact overlap, light-red = Metformin-specific pathways. (f) Correlation of gene expression changes to the aging process in-vitro in untreated and Metformin-treated cultures showing anti-aging effects.

Building on the observed heterogeneity and the prominent role of downregulated pathways in driving aging, we next examined how anti-aging treatments influence these pathways. Metformin and rapamycin are often viewed as the two most promising anti-aging drugs, as they extend lifespan in various animal models^34,35^. In humans, metformin has been shown to have slowing effects on epigenetic clocks and aging-related gene pathways and a large clinical trial is ongoing, while the impact of rapamycin on human aging remains less clear^36,37^. Establishing the efficacy of these drugs in humans remains incomplete and has already required decades of trials and billions in research investment - a resource-intensive approach that underscores the urgent need for predictive human aging models that can advance therapeutic development. To assess predictive power of our accelerated in-vitro aging model, we long-term treated cultures of n = 3 donors aged 53, 67 and 68 years with 25 nM rapamycin or 1 mM metformin over six months and performed mRNA-Seq and DNAm analysis each month. No apparent cell death or morphological changes were observed under these conditions (Fig. 4a). Interestingly, metformin treatment had a slowing effect on the DNAm and RNA age velocity, significantly reducing the average DNAm age velocity of these donors from 8.5x to 1.8x and of the RNA velocity from 48.3x to 22.0x (Fig. 4b,b, **Fig.S4c**). Also, rapamycin showed slowing trend on the DNAm and RNA clock that was however not statistically significant (**Fig.S4a-c**). To objectively assess the treatment-specific impacts on the dynamic transcriptional aging process, we performed differential expression analysis against a baseline of healthy aging. This analysis identified 335 significantly upregulated and 250 significantly downregulated genes with Metformin treatment, while Rapamycin affected only 67 upregulated and 147 downregulated genes (Fig. 4d, **Fig.S4d**). KEGG pathway analysis revealed that most of the pathways modulated by Metformin overlapped with those implicated in the aging process, whereas Rapamycin showed minimal overlap (Fig. 4e, **Fig.S4e**). We next focused on the 67 transcriptomic aging pathways that overlap between in-vivo and in-vitro aging, which showed that a majority of up-regulated pathways were slowed in response to both drugs, while only metformin had a positive slowing effect on most pathways down-regulated during aging (Fig. 4f, Fig.S4f). Rapamycin even increased the down-slope of many of these pathways, as it showed no significant changes on the pathway level, and also did not result in a lasting increased expression of autophagy-related genes over the time course. Overall, these data are consistent with data obtained from human studies where metformin showed beneficial effects on several aging-related readouts including clocks^38,39^. This indicates that prolonged treatment of in-vitro aging fibroblasts can serve as a predictive model for evaluating potential new anti-aging compounds relevant to human aging on a personalized level. The approach further appears powerful to identify aging pathways that escape specific treatments to inform about potential combinatorial approaches targeting these missed pathways.

## DISCUSSION

Collectively, this longitudinal transcriptomic and epigenomic characterization of in-vitro aging of human fibroblasts from adult donors provides novel insights into the biological aging process during extended in-vitro cell cultures. By comparing identical cell types in-vivo and in-vitro, our findings reveal that the aging process in-vitro closely mirrors many aspects of transcriptomic and epigenomic aging. Further, we observe a pronounced acceleration of aging clocks within the in vitro environment. Consequently, this phenomenon allows for the replication of multi-year time frames within just a few months in culture, presenting a unique opportunity to simulate the crucial time period preceding the onset of age-related diseases^40^. Interestingly, transcriptomic aging accelerated to several times faster speeds than DNAm aging. The substantial differences amongst transcriptomic pathway acceleration may stem from the absence of the instructive signals provided by neighboring cells and tissues in the in-vitro culture context, which differentially affects individual aging pathways. These observations indicate that the biological aging process operates at variable speeds depending on contextual cues. This fibroblast in-vitro aging model is further relevant as a donor-specific human accelerated aging model that is useful for the evaluation of pharmacological putative anti-aging interventions that are distinct from accumulation of senescent cells. Because confluent fibroblast cultures are contact-inhibited, they can be cultured for months in a non-dividing G0-like state that circumvents senescence. Further, single cell transcriptome analysis detected a substantial heterogeneity of cell ages within primary cell populations. A similar phenomenon has been observed in-vivo^30–33^, and our data further exemplifies that the increase of biological ‘noises’ is a multi-parallel and defining theme of biological aging. Our single cell data extend this notion from the intra-cell (transcriptomic, epigenetic) to inter-cell noise within a population.

Furthermore, our data have significant implications for enabling dynamic aging research using direct reprogramming models, such as iNs that retain the aging signatures of their original cell types^5,24–26,41^. Because iNs, unlike iPSCs, retain the aging characteristics of their starting cells, we have demonstrated a novel approach to generate pseudo-longitudinal aged neuronal samples by applying our in-vitro aging model to human fibroblasts and subsequently converting them into iNs. This method provides a valuable tool for studying the progressive effects of aging on neurons, potentially accelerating research into age-related neurodegenerative disorders and the development of therapeutic interventions.

Collectively, our findings suggest that in vitro aging of starting fibroblasts can be employed to ‘pre-age’ these cells prior to neuronal conversion to generate ‘pseudo-longitudinal’ neuron samples from a single donor, particularly in scenarios where longitudinal biopsies are not feasible.

## LIMITATIONS OF THE STUDY

While our in-vitro aging model offers valuable insights, it is important to recognize that it does not fully replicate the complexities of real longitudinal studies. For instance, our approach does not account for the intricate environmental factors that influence aging in-vivo, such as diet, physical activity, and exposure to various stressors. Additionally, it cannot capture patient-specific events during the aging process, including illnesses or lifestyle changes, which may significantly impact individual aging trajectories. Despite these considerations, fibroblasts provide a robust starting point for several direct conversion protocols to various cell types. Currently, our method is primarily focused on generating neurons; however, there is great potential for further development. By expanding direct conversion protocols to include alternative cell types, such as glial cells, we can enhance the capabilities of our model system. This expansion will allow for a more comprehensive exploration of cellular aging across different tissue types and contribute to a deeper understanding of age-related processes.

## STAR METHODS

### RESOURCE AVAILABILITY

#### Lead contact

Further information and requests for resources and reagents should be directed to and will be fulfilled by the lead contact Jerome Mertens (jmertens@ucsd.edu)

#### Materials availability

This study did not generate new unique reagents.

#### Data and code availability

All raw data will be uploaded to appropriate repositories upon acceptance of manuscript. Any additional information required to reanalyze the data reported in this paper is available from the lead contact upon request.

### EXPERIMENTAL MODEL AND SUBJECT DETAILS

The cohort consists of 4 fibroblast lines derived from all female donors between 53 and 68 years (**Fig.S1a**). All experiments conducted were within the approval of the institutional ethics committee. In particular, human skin samples were obtained with the ethical approval of the ethics committee of the Medical University of Innsbruck (EK Nr: 1053/2018), and informed and signed consent was obtained from the donors before collection of tissues.

#### Isolation and culture of primary human skin fibroblasts

Primary cells were isolated from skin punch biopsies, which were immediately digested using 1mg/mL Collagenase and 1mg/mL Dispase at 37 °C overnight, before enabling outgrowth of fibroblasts on Gelatin-coated wells. Pure fibroblast cultures were generated from four donors (female, 53,55,67,68 years old) and maintained in fibroblast media containing DMEM (ThermoFisher) + 15 % fetal bovine serum (ThermoFisher) + 1x non-essential amino acids (Sigma Aldrich) + 1x antibiotic-antimycotic (ThermoFisher). Cells were expanded by detaching using TrypLE (ThermoFisher) and seeded in a 1:2 ratio. Month 0 of the experiment was determined once all the lines were available in 18 separate flasks and confluent (Donor #1 and Donor #4: passage 8, Donor #2 and Donor #3: passage 9). In-vivo aging fibroblasts (passage <10) and in-vitro aging fibroblasts were harvested once reaching confluency using TrypLE and pellet at 4 °C. Pellets were stored in liquid nitrogen until processing. In-vitro aging fibroblasts were harvested each month (30 days) for six months. Metformin (1mM) and Rapamycin (25 nM) treatments were applied the day after harvesting Month 0 in fibroblast media.

### METHOD DETAILS

#### DNA/RNA Extraction

Frozen cell pellets of Months 0–6 were thawed on ice, and DNA and RNA were extracted simultaneously from the same pellet using the Quiagen AllPrep DNA/RNA Mini Kit. DNA and RNA concentrations were assessed using Qubit Fluorometric Quantification. Quality of RNA was further assessed using the TAPE Station System (Agilent), and DNA was run on an Agarose Gel.

#### Bulk RNA sequencing

RNA samples were frozen and shipped on dry-ice. In-vitro aging samples were sequenced paired-end 150bp on the NovaSeq X Plus at 20 million paired reads per samples (NovoGene) and the in-vivo aging samples were sequenced paired-end 150bp on the NextSeq at 20 million paired reads per sample. Read trimming was performed using TrimGalore and mapped with STAR to the hg38 before generating raw counts using featureCounts and Fragments Per Kilobase of transcript per Million mapped reads (fpkm) normalization. To assess RNA Age we applied the multi-tissue transcriptomic age calculator open source software ‘RNAAgeCalc’^16^ to raw counts according to the Bioconductor script. Linear Regression and correlation to age (Month 0–6, or 22–92 years of age) were calculated for the in-vitro dataset and the in-vivo dataset. All genes significantly correlating to the time-period in culture/in-vivo aging were termed “aging-genes”. KEGG pathways of genes contributing to in-vitro and in-vivo aging were determined using the Cytoscape tool ClueGO^42^. Cutoff was based on %Genes adjusted to input size, and 30 % was set as a threshold to be considered an overlapping pathway (50 % being an equal distribution between in-vitro and in-vivo aging, and 100 % being exclusively expressed in either of the groups). Representative pathways of each category were chosen based on in-vitro and in-vivo aging overlap, and genes contributing the pathway were extracted from ClueGO. An average of fpkm expression for each sample was calculated per pathway and linear regression across the in-vitro aging time-line or in-vivo aging years of aging was calculated using GraphPad Prism.

#### DNA methylation EPIC array

DNA methylation assays were performed using the Infinium Methylation EPIC v2.0 Kit, loading 8 samples per BeadChip as per the standard manufacturer’s protocol (Illumina). Raw intensity IDAT files were processed and analyzed using the R packages ChAMP and RnBeads, arrays were normalized using the BMIQ procedure. Beta-values were annotated to the EPICv2.0 annotation file and linear regression and correlation across time was calculated. Promoter regions were defined as TSS200, TSS1500, 5’UTR, and 1^st^ exon based on previous reports^23^.

#### Biological Age Estimators

Epigenetic Clock analysis was performed using the UCLA online tool ‘DNA methylation Age Calculator’ to predict chronological ages of cell culture samples^11^, and the multi-tissue transcriptional age calculator RNAAgeCalc^16^ was used to calculate RNA Age. Aging velocity based on these clocks was calculated based on the slope of linear regression (GraphPad Prism) calculated on the actual chronological age gain during aging (in-vivo aging 0–92 years, in-vitro aging 55, 55.083, 55.17, …). Years aged in a certain time period was a subtraction of estimated biological age at month 5 of biological age at month 0.

#### Direct conversion of pre-aged fibroblasts into induced neurons

Fibroblasts at starting passage were transduced with an all-in-one lentiviral vector for dox-inducible expression of the neuronal transcription factors (Ngn2 and Ascl1) and selected and expanded under puromycin. At month 0 (3–4 weeks following lentiviral transduction) and after 6 months, the transgenic fibroblasts were converted to iNs using our previously described protocol^2,24,43^. Briefly, the cells were treated with dox along with small molecule enhancers to promote neuronal identity for 3 weeks. To purify iNs, cells were dissociated using Trypsin and labeled with PSA-NCAM antibodies. Fluorescently labeled cells were sorted using FACS to isolate PSA-NCAM-positive iNs, ensuring a highly enriched neuronal population (>90% purity). Sorted cells were immediately pelleted and processed for DNA and RNA collection as described for fibroblasts.

#### Single cell RNA sequencing

Before reaching month 5 of the experiment, we thawed previously frozen batch of fibroblasts of Donors #3 and #4, representing month 0 once reaching confluency. Fibroblasts from month 0 and month 5 were simultaneously harvested for single cell RNA sequencing. Cells were counted in PBS + 0.04 % BSA and loaded on the 10x Chromium Controller targeting 8000 cells per sample. Library was prepared using the 10× 3’scRNA v3.1 library kit and 10x Dual Index Kit, and was sequenced on the NextSeq 2000 (200 cycles kit). Raw files were processed using Cell Ranger and analyzed using Seurat. Pseudobulk of each cluster was calculated as an input for RNAAgeCalc biological age estimation. Aging score was calculated per cell based on up- and down-regulated genes of the RNAAgeCalc, and LTA score was calculated based on the overlapping aging genes from in-vitro and in-vivo aging.
